# The Effectiveness of Coronary Artery Bypass Grafting in Patients with Left Ventricular Dysfunction

**DOI:** 10.21470/1678-9741-2021-0032

**Published:** 2023

**Authors:** Salih Salihi, Halil İbrahim Erkengel, Hakan Saçlı, İbrahim Kara

**Affiliations:** 1 Department of Cardiovascular Surgery, Sakarya University, Faculty of Medicine, Sakarya, Turkey.

**Keywords:** Coronary Disease, Coronary Artery Bypass, Left Ventricular Dysfunction, Quality of Life, Mortality, Intensive Care Unit

## Abstract

**Introduction:**

Coronary artery bypass grafting (CABG) in patients with left ventricular dysfunction (LVD) remains a surgical challenge and is still controversial. The aim of this study was to evaluate the effectiveness of CABG in patients with LVD.

**Methods:**

This retrospective study included a total of 160 consecutive patients (133 males, 27 females, mean age 62.1±10.12 years [range 37 to 86 years]) who had a left ventricular ejection fraction (LVEF) ≤ 45% determined by echocardiography and underwent elective isolated CABG between September 2013 and December 2018. Preoperative echocardiographic data, such as ejection fraction, left ventricular (LV) end-systolic diameter, and LV end-diastolic diameter, were collected and evaluated. Preoperatively, 85 (53.13%) patients were in New York Heart Association functional class III or IV and the mean LVEF was 38.65±5.72% (range 20 to 45).

**Results:**

The overall hospital mortality was 5% (eight patients). Late follow-up was obtained in 152 (90%) cases (median follow-up time was 56,5 [3-87] months postoperatively). During follow-up, mortality developed in 11.3% (16 patients). Mean LVEF increased significantly from 38.78±5.59% before surgery to 43.29±8.46% after surgery (*P*<0.01). Mean late survival, freedom from coronary reintervention, and congestive heart failure rates were 86.3±3.3%, 88.7±3.9%, and 89.4±3.1%, respectively.

**Conclusion:**

In patients with LVD, CABG can be performed with low postoperative morbidity and mortality rates. Patients with LVD could benefit from coronary bypass surgery regarding postoperative LV systolic function and higher quality of life.

## INTRODUCTION

Preoperative left ventricular dysfunction (LVD) is a well-known risk factor for early and late mortality after coronary artery bypass grafting (CABG), and the management of these patients is still a difficult and challenging issue^[1]^. As a result of recent advances in perioperative anesthesia management, surgical techniques, myocardial protection methods, and postoperative care, postoperative results have been improved, and the number of patients with LVD referred for CABG is on the rise^[2]^. The effectiveness of CABG in LVD patients is variable; some studies have found ventricular improvement early after operation^[3]^, while others have determined no change^[4,5]^ or a deterioration of left ventricular (LV) function^[6]^. Different series assessing the efficacy of surgical revascularization in patients with LVD have demonstrated that they benefit from revascularization especially if symptoms of angina or ischemia are present^[7]^. Coronary revascularization surgery in patients with LVD preserves the residuary viable myocardial tissue, prevents additional myocardial degeneration, and provides the improvement of systolic function of ischemic and hibernated myocardial segments^[8]^. The purpose of this study was to evaluate the effectiveness of CABG in patients with LVD who underwent isolated CABG in our hospital.

## METHODS

### Patients

From September 2013 to December 2018, 874 patients underwent isolated CABG in our institution. This retrospective study included a total of 160 consecutive patients (133 males, 27 females, mean age 62.1±10.22 years [range 37 to 86 years]) who had a left ventricular ejection fraction (LVEF) ≤ 45% determined by echocardiography and underwent elective isolated CABG in that period. Patients who had LV aneurysm, prior CABG, or combined CABG with other valve interventions were excluded from this study. Indications for bypass surgery were based on standard clinical and angiographic criteria.

All preoperative, operative, and postoperative data were collected. Surgical and discharge notes were reviewed. A written informed consent was obtained from each patient. The study protocol was approved by the local Ethics Committee (nº 71522473/050.01.04/12, date: 27/01/2020) and was conducted in accordance with the principles of the Declaration of Helsinki.

### Surgical Methods

Standard anesthetic technique was used during induction (fentanyl, midazolam, and pancuronium) followed by the maintenance of isoflurane and propofol. All operations were performed via median sternotomy. The internal thoracic artery (ITA) and saphenous vein graft was prepared if necessary. Surgical revascularization was performed under cardiopulmonary bypass (CPB) (except thirteen patients). CPB was established via standard aortic arterial and two-stage venous cannulation. Antegrade cardioplegia delivery cannulas were inserted into the aortic root. In selected patients (left main lesions and acute coronary syndromes), the retrograde cardioplegia cannulas were inserted into the coronary sinus in addition to antegrade cannulas. Diastolic arrest was maintained by delivery of intermittent, moderately hypothermic blood cardioplegia in all patients. Body temperature was maintained between 28°C and 30°C during CPB. Distal anastomoses were performed under aortic cross-clamping while proximal anastomoses were performed with side clamping during rewarming. ITA was routinely applied for left anterior descending artery revascularization, and saphenous vein graft was anastomosed to other target vessels. Before removal of cross-clamp, a last cardioplegic solution (hot-shot) at 37°C was delivered.

### Follow-up

Follow-up was achieved via monthly periodical examinations in the first three months, and thereafter by either regular cardiology visits or phone contact. In some patients, echocardiography was performed during follow-up when necessary. The median follow-up time for all patients was 56,5 (3-87) months. Preoperative transthoracic echocardiography (TTE) was obtained, as well as TTE was performed during follow-up. When we started this study, echocardiography was performed by calling the patients whom we could reach. Ejection fraction is commonly measured by echocardiography, in which the volumes of the heart’s chambers are measured during the cardiac cycle. Other echocardiographic parameters (left ventricular end-systolic diameter [LVSD], left ventricular end-diastolic diameter [LVDD], mitral regurgitation, and tricuspid regurgitation) were assessed. After CABG, statins, antiplatelet agents, renin-angiotensin-aldosterone system inhibitors (angiotensin-converting enzyme inhibitors or angiotensin II receptor blockers), and β-blockers were given to all patients with no contraindications.

### Statistical Analysis

Data analysis was performed by using IBM Corp. Released 2017, IBM SPSS Statistics for Windows, Version 25.0, Armonk, NY: IBM Corp. The variables were investigated using visual (histograms, probability plot) and analytical (Kolmogorov-Smirnov/Shapiro-Wilk’s test) methods to determine whether they are normally distributed. The continuous variables were expressed as mean and standard deviation or as median and interquartile range, depending on the normality of their distribution. In two different periods, the paired samples *t*-test was preferred to compare parametric variables. The statistically significant two-tailed *P*-value was considered as < 0.05. Actuarial estimates for cumulative survival and freedom from adverse events were calculated using the Kaplan-Meier method.

## RESULTS

Baseline characteristics and comorbidities of the study population are presented in Table 1. Their mean age was 62.1±10.22 (range 37 to 86) years. Mean preoperative LVEF was 38.65±5.72% (range 20 to 45), and 27 (16.9%) patients were female. A total of 85 patients (53.13%) were in the New York Heart Association (NYHA) functional class III-IV. Twenty patients (12.5%) had moderate mitral regurgitation, and two patients (1.3%) had moderate tricuspid regurgitation.

**Table 1:** Demographic characteristics.

Preoperative variable	N=160
Age (years)	62.1±10.22
Sex (female)	27 (16.9%)
Hypertension	107 (66.9%)
Diabetes mellitus	79 (49.4%)
Peripheral artery disease	15 (9.4%)
Carotid artery disease	18 (11.3%)
Smoking	64 (40%)
Renal failure	5 (3.1%)
NYHA class	
I, II	75 (46.87%)
III, IV	85 (53.13%)
Number of involved vessels	
Single-vessel disease	9 (5.6%)
Two-vessel disease	49 (30.6%)
Three-vessel disease	102 (63.7)
LMCA disease in catheterization	37 (23.1%)
Echocardiographic parameters	
LVEF (%)	38.65±5,72
LVSD (mm)	38.03±5.67
LVDD (mm)	50,72±5.72
Moderate mitral regurgitation	20 (12.5%)
Moderate tricuspid regurgitation	2 (1.3%)

Data are presented as mean value ± standard deviation or number of patients and percentage. Diabetes mellitus: insulin dependent or not.

LVDD=left ventricular end-diastolic diameter; LVEF=left ventricular ejection fraction; LVSD=left ventricular end-systolic diameter; LMCA=left main coronary artery; NYHA=New York Heart Association

One hundred and two (63.7%) patients had three-vessel disease, followed by two-vessel disease (30.6%), and single-vessel disease (5.6%). There were 37 (23.1%) patients with left main coronary artery lesion. The number of patients who underwent conventional CABG was 144 (90%), and off-pump CABG was performed in 16 (10%) patients. The mean number of grafts per patient was 2.5±0.8. We have performed coronary artery endarterectomy in 18 (11.3%) patients. The operative data have been summarized in Table 2.

**Table 2:** Intraoperative parameters.

Variables	N=160
Surgical approach	
On-pump	144 (90%)
On-pump beating heart	3 (1.9%)
Off-pump	13 (8.1%)
Cardioplegia	
Antegrade	122 (76.3%)
Antegrade and retrograde	22 (13.8%)
None	16 (10%)
Use of ITA	138 (86.3%)
Vessel quality	
Good	88 (55%)
Very plagued	72 (45%)
Number of grafts used (mean±SD)	2.5±0.8
Coronary artery endarterectomy	
LAD artery	18 (11,3%)
Sequential anastomosis	8 (5%)
ICU stay, days (median)	2 (1-25)
Hospital stay, days (median)	7 (2-33)

Data are presented as mean value ± standard deviation, median value (minimum-maximum), or number of patients and percentage.

ICU=intensive care unit; ITA=internal thoracic artery; LAD=left anterior descending artery; SD=standard deviation

### Early and Late Outcomes

The early and late postoperative outcomes of all patients are presented in Table 3. Overall in-hospital mortality rate was 5% (n=8). Three patients (1.87%) had low cardiac output, one patient had sepsis, and one (0.6%) died of acute renal failure. Twenty-one (13.1%) patients required intra-aortic balloon pump support. Five patients (3.1%) had cerebrovascular events, and 16 (10%) had renal failure in the postoperative period.

**Table 3:** Early and late morbidity and mortality.

Variables	
Early (< 30 days)	N=160
Mortality	8 (5%)
Early complications	
Low cardiac output syndrome	19 (11.9%)
Intra-aortic balloon pump	21 (13.1%)
Inotropic support > 24 hours	41 (25.6%)
New-onset atrial fibrillation	37 (23.1%)
Reoperation for bleeding	9 (5.6%)
Pleural effusion requiring drainage	17(10.6%)
Postoperative renal failure*	16 (10%)
Hemodialysis	7 (%)
Cerebrovascular accident	5 (3.1%)
Pulmonary complications	41 (25.6)
Superficial wound infection	2 (1.3%)
Late	N=152
Mortality	16 (10.5%)
Cardiac	4 (2.6%)
Non-cardiac	10 (6.6%)
Unknown	2 (1.3%)
Coronary reintervention	9 (5.9%)
Heart failure (NYHA III, IV)	11 (7.2%)

Data are presented as number and percentage

* Creatinine level of > 2 mg/dl

NYHA=New York Heart Association

The patients’ median intensive care unit stay was two days (range 1 to 25), and the median in-hospital stay was seven days (range 2 to 33).

Late follow-up data were obtained in 152 (90%) cases (median follow-up time was 56,5 [3-87] months postoperatively). Four of them (2.6%) died from cardiac causes, ten (6.6%) from other causes, and two (1.3%) from unknown causes.

Control echocardiographic evaluations (Table 4) showed that the mean postoperative LVEF (43.29±8.46%) and LVDD of patients (49.31±5.61) were significantly better than that of the preoperative period (38.78±5.59% and 50.45±5.27, respectively) (*P*-value < 0.001). Nonetheless, there were no significant changes in LVSD after CABG (*P*=0.833).

**Table 4:** Changes in postoperative echocardiographic parameters for all patients.

Echocardiographic parameters	Preoperative	Postoperative	P-value
LVEF (%)	38.78±5.59	43.29±8.46	< 0.001
LVSD (mm)	37.91±5.54	37.81±6.32	0.833
LVDD (mm)	50.45±5.27	49.31±5.61	< 0.001

LVDD=left ventricular end-diastolic diameter; LVEF=left ventricular ejection fraction; LVSD=left ventricular end-systolic diameter

**Fig. 1:**
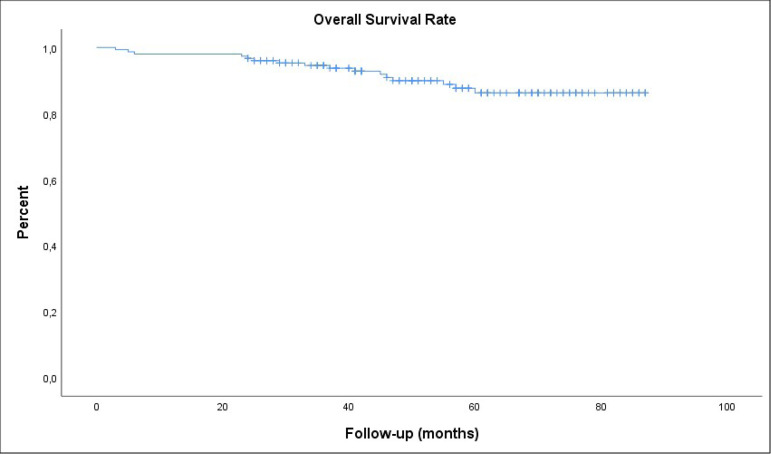
Cumulative survival analysis of the patients.

Cumulative survival analysis of the patients assessed by the Kaplan-Meier method revealed an overall survival rate of 86.3±3.3% at eight years (Figure 1).

**Fig. 2:**
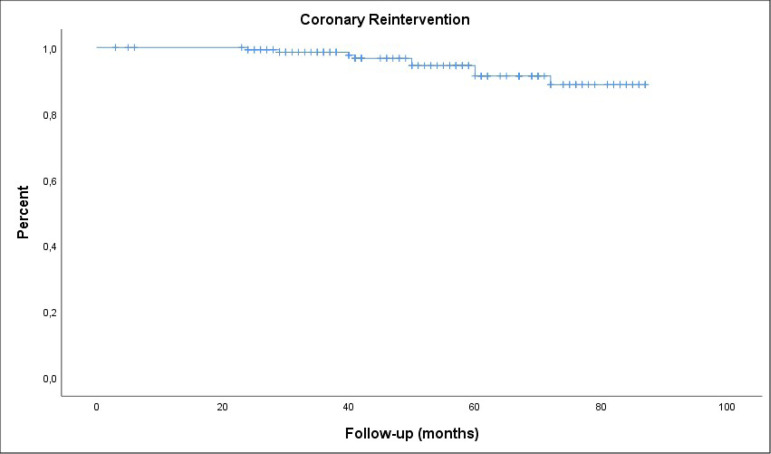
Kaplan-Meier analysis of freedom from coronary reintervention in all patients.

Nine patients (5.9%) had coronary reintervention during follow-up. Overall rate of freedom from coronary reintervention was 88.7±3.9% at eight years (Figure 2).

**Fig. 3:**
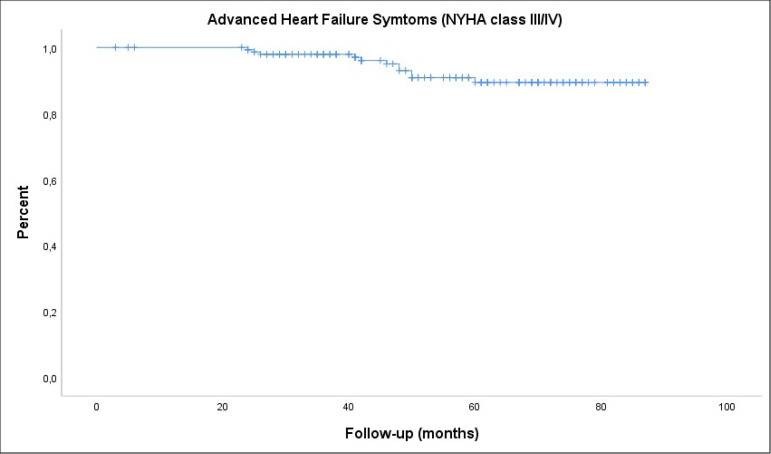
Kaplan-Meier analysis of freedom from advanced congestive heart failure in all patients. NYHA=New York Heart Association.

There were 11 patients (7.2%) in the NYHA functional class III-IV. Overall rate of freedom from congestive heart failure was 89.4±3.1% at eight years (Figure 3).

## DISCUSSION

In this study the effectiveness of CABG in patients with LVD was emphasized. A significant improvement in LV systolic function was observed after CABG in them (mean LVEF increased significantly from 38.78±5.59% before surgery to 43.29±8.46% after surgery). Kaplan-Meier survival analysis estimates were 86.3±3.3% for late survival.

Ischemic cardiomyopathy is the most common cause of heart failure which can result from hibernation, myocardial stunning, and from scar tissue following myocardial infarction^[9]^. Patients with significant coronary artery disease (CAD) and LVD are a high-risk patient population, and in these patients the decision to undergo revascularization can be challenging.

One of the persisting challenging areas of treatment for a cardiac surgeon is curing a patient with LVD. The STICH trial and its extension study (STICHES) evaluated the role of CABG and optimal medical therapy (OMT) *vs*. OMT alone in 1,212 patients with stable significant CAD and LVD (mean LVEF 28%). CABG combined with OMT is associated with higher risk of short-term mortality but improved long outcomes in these patients compared with medical therapy alone^[10,11]^. In the CASS trial, it has been reported that CABG provides a better prognosis compared with medical therapy in patients with LVD^[12]^. Furthermore, with the growing number of patients with LVD requiring coronary artery revascularization, the best method of revascularization is still a matter of debate. The 2018 European Society of Cardiology and the European Association of Cardio-Thoracic Surgery Guidelines are the most up to date^[13]^. They recommend revascularization for CAD and LVD (LVEF ≤ 35%; class I, LOE B) and CABG as the preferred revascularization strategy for multivessel CAD and acceptable surgical risk (class I, LOE B). In addition, Mark et al.^[14]^ reported that CABG was associated with improvements in health-related quality of life (QOL) outcomes compared with OMT alone. In patients with significant CAD and LVD, CABG is superior to percutaneous coronary revascularization in terms of long-term survival and freedom from repeat revascularization in several observational studies^[11,12]^. Yanagawa B et al.^[15]^ reported that surgical revascularization confers a long-term survival benefit in these patients.

As one of therapeutic aims of coronary revascularization, improvement in systolic function has been accepted as the reference standard for the assessment of myocardial viability^[16]^. The role of assessment of myocardial viability in LVD patients before surgical revascularization remains controversial. According to the recent study published by Julio A Panza et al.^[17]^, there is no relationship between myocardium viability and long-term benefit of CABG in patients with LVD. But they reported that the presence of viable myocardium was associated with improvement in LV systolic function. In our center, we assessed patients before CABG routinely with TTE. However, if the patient has not graftable coronary arteries in coronary angiography and there is a doubt about myocardium viability, we use myocardial perfusion scan thallium to evaluate myocardium viability.

CABG with CPB has been reported to carry several risks for patients with LVD^[18]^. And although the reported benefits of off-pump CABG, its influence in patients with LVD still controversial^[19]^. According to Attaran et al.^[20]^, there was no difference with or without CPB in terms of operative complications, hospital mortality, and short-, mid-, and long-term survival rates in patients with LVD. Toumpaulis et al.^[21]^ reported that off-pump CABG had early and midterm outcomes like those of on-pump CABG in patients with LVD. We preferred off-pump CABG in 16 (10%) patients with single-vessel disease.

Successful myocardial protection and complete revascularization are important hints and predictors of favorable short- and long-term results after CABG in patients with LVD^[22]^. Attaran et al.^[20]^ reported that there was no significant influence of CPB on the in-hospital mortality, midterm survival, or long-term survival in LVD patients. The most important issues were adequate myocardial protection and complete revascularization. We used multidose antegrade cardioplegia alone in most patients, and combined antegrade and retrograde cardioplegia in patients with coronary total occlusion. Complete revascularization was our aim in all patients.

Patients with CAD and impaired LV function represent a high-risk group referred for CABG. In our study, the overall hospital mortality was 5% (eight patients), and there were 16 (10.5%) late mortalities. Our mortality rates are similar to that reported by Islamoğlu et al.^[23]^ (5.13% and 16.12%, respectively).

Most of our patients had significant improvement of LVEF in the postoperative control echocardiographic evaluations as the mean LVEF increased significantly from 38.78±5.59% before surgery to 43.29±8.46% after surgery. In the study reported by Khaled S et al.^[24]^, there was a significant improvement in LVEF after surgery (from 29.76±4.86 before surgery to 33.53±9.65 after surgery). In another study, LVEF increased from 25.6±5.2 to 31.08±5.5, postoperatively. The authors reported a significant improvement in both angina and heart failure status^[25]^.

### Limitations

This study has several limitations. Firstly, the small patient population and the retrospective nature of this study do not allow us to draw satisfactory conclusion about the effectiveness of CABG in patients with LVD. Secondly, the follow-up periods were limited in some cases. Thirdly, the results of this study are encouraging, and it needs corroboration in multicenter larger populations with longer follow-up. Moreover, there was no specific time frame to perform the postoperative echocardiography, and this could interfere with the evaluation of LV restoration. Finally, medication use was not systematically collected, but prescribed for all patients.

## CONCLUSION

CABG can be safely performed in patients with LVD with low postoperative morbidity and mortality rates. LVD patients could benefit from coronary bypass surgery regarding postoperative LV systolic function and higher QOL.
